# Institutional Questions

**Published:** 1900-04-21

**Authors:** 


### INSTITUTIONAL QUESTIONS.

### Sanatoria for Consumptives.

A correspondent asks: Can you tell me where plans of recently-built sanatoria, especially for consumptives, with a view to carrying on the open-air treatment in this country, may be seen, if any have been published?

*** You will probably find what you require in a book published a short time since entitled "Sanatoria for Consumptives," by Dr. R Watters (Sonnenschein). Plans and much information on this subject will be found in this book. Also during last year the *Practitioner* and the *West London Hospital Gazette* devoted a certain amount of space to this question, and a study of their back numbers might help you.

### A Uniform System.

"Provincial Secretary" writes: I understand that the "Uniform System of Accounts" advocated for hospitals has been now practically adopted for all London hospitals. Will you kindly inform me (I have not a practical acquaintance with the system myself) whether it can be adapted to institutions other than hospitals, or if a modified form can be procured for use in such institutions?

*** In the chapter on "General Charities" in the last edition of "Hospitals and Charities" (1899) you will find this matter discussed at some length. It is there urged that, following the example of hospitals, the managers of the various classes of charities should meet in consultation and evolve a suitable system for keeping their accounts which should conduce to the advantage of all. Failing this united action, you would nevertheless do well to study the book issued by the Scientific Press, "The Uniform System of Accounts, Audit, and Tenders for Hospitals and Institutions." Forms are given suitable to the requirements of large or small institutions, and complete sets of account books, embodying the various forms, are also to be procured. The editor will, if consulted, gladly make modifications in the forms to suit special institutions. The fact that the hospitals are surely, if slowly, recognising the advantages offered by a uniform system, whereby comparisons may be made with fairness and accuracy, shows that the system works well, and thoroughly justifies the claims made on its behalf.

